# Machine Learning–Based Classification of Depression Using Inflammatory Biomarkers in Pancreatic Cancer Patients

**DOI:** 10.1002/kjm2.70094

**Published:** 2025-08-24

**Authors:** Yang‐Chen Shen, Po I Wu, Cheng‐Feng Lin, Chia‐Jui Yen, Yan‐Shen Shan, Po See Chen

**Affiliations:** ^1^ Institute of Behavioral Medicine College of Medicine, National Cheng Kung University Tainan Taiwan; ^2^ Department of Medicine College of Medicine, National Cheng Kung University Tainan Taiwan; ^3^ Department of Physical Therapy College of Medicine, National Cheng Kung University Tainan Taiwan; ^4^ Department of Oncology National Cheng Kung University Hospital, College of Medicine, National Cheng Kung University Tainan Taiwan; ^5^ Institute of Clinical Medicine College of Medicine, National Cheng Kung University Tainan Taiwan; ^6^ Department of Surgery National Cheng Kung University Hospital, College of Medicine, National Cheng Kung University Tainan Taiwan; ^7^ Department of Psychiatry National Cheng Kung University Hospital, College of Medicine, National Cheng Kung University Tainan Taiwan

**Keywords:** depression, inflammatory biomarkers, machine learning, pancreatic cancer

## Abstract

Inflammation is a common mediator of pancreatic cancer and depression. This study investigated the predictive value and clinical associations of inflammatory markers and depression in cancer patients using machine learning (ML) and statistical modeling. Pancreatic cancer patients (*n* = 328; mean age, 65 years; majority with stage IV disease) were assessed using the Patient Health Questionnaire‐9 (PHQ‐9; depression defined as PHQ‐9 ≥ 10). Clinically significant depression was present in 35% of subjects at baseline, and the rate declined at follow‐up. Four ML models (logistic regression, random forest, support vector machine, and extreme gradient boosting; XGBoost) were trained using routinely collected clinical data and showed comparable performances with moderate but consistent discriminative capacity (AUC: 0.70–0.72). Permutation importance analysis revealed C‐reactive protein (CRP), neutrophil‐to‐lymphocyte ratio (NLR), and albumin as key predictors of depression. Generalized estimating equations further confirmed that elevated CRP (OR = 1.32, *p* = 0.001) and NLR (OR = 1.55, *p* = 0.001) were independently associated with depression. These findings suggest that inflammatory markers can not only help to identify patients at risk for depression but also underscore the linkage between inflammation and depression. ML models incorporating these markers may therefore support early detection and intervention in pancreatic cancer care.

## 1 Introduction

Pancreatic cancer is one of the most lethal malignancies worldwide and is widely known for its high mortality rates and late‐stage diagnosis [1]. Despite advancements in cancer therapies over the past two decades, the five‐year survival rate for pancreatic cancer is only around 10% [2], and it is the seventh leading cause of global cancer mortality [3]. Increasing incidence and death rates for the disease underscore the urgent need for improved understanding, prevention, and treatment strategies [4].

The clinical profile of pancreatic cancer is complicated by a high prevalence of depression, which far exceeds that in the general population [5, 6, 7]. Depression in pancreatic cancer patients is linked to worse outcomes, including lower quality of life, poorer treatment compliance, and reduced survival [8, 9, 10, 11, 12, 13]. Despite these significant impacts, depression in pancreatic cancer has received little attention, and further investigations into its prevalence, causes, and effects are needed.

Depression may precede pancreatic cancer diagnosis and serve as an early warning sign of disease, highlighting the complex interplay between mental health and cancer [14]. At the molecular level, inflammatory cytokines are pivotal to both conditions and play a critical role in their co‐occurrence [15]. Moreover, the relationship between inflammation and depression appears to be bidirectional, with cytokines potentially contributing to the development of depression, and depression in turn amplifying inflammatory processes [16]. Thus, depression in pancreatic cancer patients may not simply be the result of psychosocial stressors and might be driven in part by biological alterations triggered by the cancer itself. Additionally, psychoneuroimmunology studies have demonstrated that prolonged distress and depression can compromise immune defenses within the tumor microenvironment via mechanisms linked to the sympathetic nervous system [17, 18].

Recent studies have applied machine learning (ML) to predict depression based on measurements of inflammatory markers [19]. A growing body of literature highlights the expanding role of ML in mental health, detailing its application in diagnosis, monitoring, and intervention for psychiatric conditions [20]. Nevertheless, it remains unclear whether depression in pancreatic cancer can be predicted from inflammatory biomarkers using ML. By harnessing the unparalleled ability of ML to uncover discreet associations, researchers may be able to identify clinically relevant inflammatory signatures that predict depression in pancreatic cancer, enabling early detection and targeted interventions. This interdisciplinary approach combines insights from oncology, psychiatry, and artificial intelligence, illustrating how technological advancements and a deep understanding of disease mechanisms can be integrated to improve patient outcomes in the evolving landscape of cancer care [21].

## 2 Materials and Methods

### 2.1 Participants

A total of 328 pancreatic cancer patients were prospectively recruited from National Cheng Kung University Hospital (NCKUH, Taiwan) between May 2021 and November 2023. Eligible patients **(**≥ 20 years old, ICD‐10 confirmed diagnosis**)** provided written informed consent, and the study was approved by the IRB (B‐ER‐110‐060). Treatments, including chemotherapy, radiotherapy, and surgery, were administered based on clinical judgment. A prospective, repeated‐measures design was implemented; data were collected at baseline (pre‐treatment) and 2, 3, 4, 6, 9, and 12 months post‐treatment.

### 2.2 Measures

#### 2.2.1 Demographic and Clinical Characteristics

Patient demographic and clinical data were obtained from electronic health records (EHR) at NCKUH. Parameters included age, sex, body mass index (BMI), cancer stage, receipt of surgical resection, chemotherapy, and radiation therapy.

#### 2.2.2 Patient Health Questionnaire‐9 (PHQ‐9)

Depression severity was assessed using the Patient Health Questionnaire‐9 (PHQ‐9), a self‐report tool with scores ranging from 0 to 27. Severity levels were categorized as minimal (0–4), mild (5–9), moderate (10–14), moderately severe (15–19), and severe (20–27), with a cutoff score of ≥ 10 indicating clinically significant depression. This threshold is widely used in cancer populations, exhibiting 77% sensitivity and 94% specificity [22].

#### 2.2.3 Inflammatory Markers

Laboratory data from NCKUH medical records included C‐reactive protein (CRP), neutrophil‐to‐lymphocyte ratio (NLR), platelet‐to‐lymphocyte ratio (PLR), and albumin. CRP ≥ 10 mg/L indicates inflammation, while albumin levels typically range from 3.5 to 5 g/dL. Variance Inflation Factors (VIF) were calculated to assess multicollinearity, with values for albumin (1.54), Ln CRP (1.67), Ln NLR (1.67), and Ln PLR (1.46), confirming no severe multicollinearity issues.

### 2.3 Statistical Analysis

Descriptive statistics were used to express the demographic and clinical characteristics of the patient population. The data are reported as mean and standard deviation (SD) for continuous variables and as frequency or percentage for categorical variables. The prevalence of depression (PHQ‐9 ≥ 10) among pancreatic cancer patients was represented as a percentage.

In the analysis, continuous variables included age, body mass index (BMI), and four inflammatory markers. Because the levels of CRP, NLR, and PLR were non‐normally distributed, the values were log‐transformed using the natural logarithm (log e) prior to analysis. Initially, all variables were included in the univariate analysis. Categorical variables, numerically coded for analysis, were as follows: Gender was coded with males as 1 and females as 0, with females as the reference group. Cancer stage was coded with stage IV as 1 and stages I, II, and III as 0, with stages I, II, and III serving as the reference group. Receipt of surgical resection was coded with those who received surgical resection as 1 and those who did not as 0, with not received serving as the reference group. Receipt of chemotherapy was coded with those who received chemotherapy as 1 and those who did not as 0, with not received serving as the reference group. Receipt of radiation therapy was coded with those who received radiation therapy as 1 and those who did not as 0, with imputation of missing values performed where necessary.

A generalized estimating equation (GEE) was used to estimate the parameters (i.e., demographic and clinical variables) and to determine whether inflammatory markers (e.g., CRP, NLR, PLR and albumin) were associated with depression. The variables that exhibited statistical significance in the univariate analysis were then incorporated into a multivariate analysis. *p‐*values less than 0.05 were considered significant in all statistical analyses; all reported *p‐*values are two‐tailed. All of the above statistical procedures were conducted with SPSS version 17 software for Windows (SPSS Inc., Chicago, IL).

The dataset was initially filtered to retain only instances with complete PHQ‐9 outcomes. Missing values in predictor variables were addressed using multivariate imputation via the “IterativeImputer” from the “Scikit‐learn” package (Python 3.13.3). Imputation was performed using a K‐nearest neighbors regression model (n_neighbors = 5) as the estimator, with a maximum of 10 iterations to ensure convergence. In total, 5.59% of the data were imputed.

After imputation, the dataset was randomly split into training (70%) and testing (30%) sets. Four ML models were applied: logistic regression (LR), random forest (RF), extreme gradient boosting (XGBoost), and support vector machine (SVM). Each model was selected based on its distinctive strengths. LR offers simplicity and interpretability, making it well‐suited for clinical risk modeling. RF leverages ensemble learning with multiple decision trees to enhance accuracy and mitigate overfitting. XGBoost integrates bagging and boosting strategies to iteratively correct weak learners and improve predictive performance. SVM is effective in handling high‐dimensional feature spaces.

To mitigate class imbalances in the outcome variable (clinically significant depression), the parameter class weight = “balanced” was applied for LR, RF, and SVM models; scale_pos_weight was used for XGBoost. These adjustments were incorporated into the hyperparameter tuning process for each model.

Hyperparameters were optimized using grid search with 10‐fold cross‐validation on the training set. The performances of the best‐tuned models were then evaluated with the independent test set. The primary readout was area under the receiver operating characteristic curve (AUC), and other metrics (e.g., accuracy, precision, recall, F1‐score, and specificity) were evaluated as well.

To assess predictor importance, permutation feature importance analysis was conducted, ranking features based on their impact on model performance. All ML analyses were performed using Python 3.13.3.

## 3 Results

### 3.1 Cohort Demographics and Prevalence of Depression

The average age of the study participants was 65 years, and 181 patients (55.18%) were male. Most patients had stage IV cancer (54.9%), underwent chemotherapy (96.04%), and did not undergo surgical resection (65.5%) or radiation therapy (88.11%) (Table 1). Descriptive statistics are shown for inflammatory markers, including albumin, CRP, NLR, and PLR, at various time points. The depression rate was highest at the baseline assessment, with 62 patients (35%) exhibiting clinically significant depression (PHQ‐9 ≥ 10); lower rates were observed at follow‐up assessments (Table 2).

**TABLE 1:** Demographic and clinical characteristics of the study population.

Variable	*N*	%	Mean	SD
Age			65	10.83
Sex				
Female	147	44.82		
Male	181	55.18		
Tumor stage				
I	19	5.8		
II	44	13.4		
III	78	23.8		
IV	180	54.9		
Missing	7	2.1		
Surgery				
Yes	113	34.45		
No	215	65.55		
Chemotherapy				
Yes	315	96.04		
No	13	3.96		
Radiation therapy				
Yes	39	11.89		
No	289	88.11		

**TABLE 2:** Descriptive statistics for inflammatory markers and depression (PHQ‐9).

	Total *N*	Albumin				CRP				NLR			PLR			PHQ‐9			
		*N*	Mean	SD	< 3.5%	*N*	Mean	SD	≥ 10%	*N*	Mean	SD	*N*	Mean	SD	*N*	Mean	SD	≥ 10%
Month 0	328	287	3.89	0.56	23.0	281	30.00	48.50	47.7	287	5.93	7.61	286	229.66	175.05	177	7.73	5.93	35.0
Month 2	326	244	3.74	0.63	31.6	129	60.40	77.42	66.7	311	4.73	7.03	311	201.30	150.34	189	5.99	4.98	20.6
Month 3	313	229	3.81	0.58	28.4	157	24.66	40.57	39.5	292	4.60	7.46	292	206.67	173.76	162	6.21	4.80	19.1
Month 4	301	254	3.74	0.72	30.7	209	34.79	57.05	48.3	284	6.06	12.58	284	215.12	183.3	229	6.21	5.45	22.7
Month 6	273	218	3.73	0.66	28.9	171	34.21	54.62	42.7	253	5.12	11.97	253	206.58	153.14	207	5.88	5.68	22.7
Month 9	219	182	3.7	0.74	34.1	152	25.50	44.94	37.5	200	4.48	5.53	200	226.99	194.41	137	5.34	4.47	17.5
Month 12	163	141	3.77	0.72	30.5	121	25.46	43.26	39.7	150	4.62	5.24	150	220.82	191.41	107	5.36	4.99	19.6

*Note*: Total *N* represents the number of participants who reached each time point. Albumin was measured in grams per deciliter (g/dL).

Abbreviations: CRP, (C‐reactive protein) was measured in milligrams per liter (mg/L); NLR, neutrophil–lymphocyte ratio; PHQ‐9, Patient Health Questionnaire‐9; PLR, platelet lymphocyte ratio.

### 3.2 Associations Between Depression and Demographic or Clinical Variables

The univariate generalized estimating equation analysis revealed significant associations of depression with both the absence of surgical resection and the presence of metastatic disease in pancreatic cancer patients (*p* < 0.05). The odds ratio (OR) for the “Tumor Stage (Stage IV)” group was 1.55, indicating that patients with Stage IV pancreatic cancer were 55% more likely to experience depression compared to those with stages I, II, or III disease. For the “Received Surgery” group, the OR was 0.57, suggesting that patients who underwent surgical resection were 43% less likely to experience depression than those who did not. However, these associations were not significant in the multivariate analysis. Other demographic and clinical variables, including age, gender, BMI, receipt of chemotherapy, and receipt of radiation therapy, were not significantly associated with depression in pancreatic cancer patients (Table 3).

**TABLE 3:** Univariate and multivariate generalized estimating equations to identify factors associated with clinically significant depression (PHQ‐9 ≥ 10) in patients with pancreatic cancer.

Variable	Univariate analysis		Multivariate analysis	
	OR	*p*	OR	*p*
Age	1.00	0.923		
Gender (Male)	0.84	0.301		
BMI	1.01	0.699		
Tumor stage (Stage IV)	1.55	0.012	1.05	0.844
Received surgery	0.57	0.002	0.69	0.128
Received chemotherapy	1.68	0.386		
Received radiation therapy	0.88	0.637		
Albumin	0.46	< 0.001	0.83	0.310
Ln CRP	1.54	< 0.001	1.32	0.001
Ln NLR	1.83	< 0.001	1.55	0.001
Ln PLR	1.50	0.002	0.82	0.220

Abbreviations: CRP, C‐reactive protein; NLR, neutrophil–lymphocyte ratio; PHQ‐9, Patient Health Questionnaire‐9; PLR, platelet lymphocyte ratio.

### 3.3 Association Between Depression and Inflammatory Markers

In the univariate generalized estimating equations analysis, levels of albumin, log‐transformed CRP, NLR, and PLR were significantly associated with depression in pancreatic cancer patients (*p* < 0.05). However, in the analysis with multivariate generalized estimating equations, only log‐transformed CRP and NLR remained significantly associated with depression. The OR for Ln CRP was 1.32, indicating that each unit increase in log‐transformed CRP levels was associated with a 32% higher likelihood of experiencing depression. Similarly, the OR for Ln NLR was 1.55, indicating that each unit increase in log‐transformed NLR corresponded to a 55% increased likelihood of experiencing depression (Table 3). We further compared the inflammatory markers between patients who underwent surgery and those who did not. The results indicated that patients who did not undergo surgery had significantly higher levels of inflammatory markers (Table 4).

**TABLE 4:** Comparison of inflammation markers between surgical and non‐surgical groups.

Variable	Surgery	Mean	SD	*p*
Albumin	Yes	3.923	0.035	< 0.001
	No	3.596	0.039	
Ln CRP	Yes	1.923	0.226	0.004
	No	2.921	0.140	
Ln NLR	Yes	0.887	0.040	< 0.001
	No	1.353	0.446	
Ln PLR	Yes	5.083	0.033	0.003
	No	5.223	0.033	

Abbreviations: CRP, C‐reactive protein; NLR, neutrophil–lymphocyte ratio; PLR, platelet lymphocyte ratio.

### 3.4 Predictive Machine Learning Models for Depression

Table 5 summarizes the average performance metrics of the four ML models used for predicting clinically significant depression (PHQ‐9 ≥ 10) in patients with pancreatic cancer. The AUC values ranged from 0.70 to 0.72 across models; indicating moderate but consistent discriminative capacity with routinely collected clinical data.

**TABLE 5:** Average metrics of four models of depression in patients with pancreatic cancer.

Model	AUC	Accuracy	Precision	Recall	F1‐score	Macro F1‐score	Specificity
Logistic regression	0.72	0.67	0.38	0.66	0.48	0.62	0.68
Random forest	0.70	0.70	0.38	0.51	0.43	0.61	0.75
XGBoost	0.71	0.69	0.39	0.64	0.48	0.63	0.70
Support vector machine	0.71	0.69	0.39	0.63	0.48	0.63	0.71

*Note*: AUC, area under the receiver operating characteristic curve. Precision, recall, and F1‐score reflect performance on the positive class (clinically significant depression, defined as PHQ‐9 ≥ 10). Macro F1‐score represents the unweighted average of F1‐scores for both classes and is particularly informative in imbalanced datasets.

Precision was relatively low across models; however, recall was higher for LR (0.66) and XGBoost (0.64), reflecting better sensitivity in identifying depression cases. Given the class imbalance in the dataset, macro F1‐scores were calculated to provide a more complete evaluation of classification performance. The unweighted average of F1‐scores across classes is particularly informative for imbalanced datasets. XGBoost and SVM had the highest macro F1‐scores (both 0.63), with LR following closely (0.62), suggesting that the ML models had comparable performance in evaluating cases with and without depression. Specificity reflects the ability to correctly identify patients without depression. This parameter was highest for RF (0.75), followed by SVM (0.71) and XGBoost (0.70), suggesting that these models could effectively minimize false positives.

Subsequent permutation importance analysis was performed to rank features based on their significance in predicting depression across multiple ML models (Table 6). The numerical values in Table 6 represent the importance rankings of each feature, with a rank of “1” indicating the highest importance. According to the permutation importance analysis, CRP, NLR, and albumin were identified as the most significant predictors of depression in pancreatic cancer patients across multiple ML models. CRP consistently emerged as a top predictor, holding the highest rank for the LR and RF models.

**TABLE 6:** Feature importance rankings across machine learning models in predicting depression in patients with pancreatic cancer.

Features	Rank		
	Logistic regression	Random forest	XGBoost
Age	5	4	5
Gender	7	6	8
BMI	9	5	4
Tumor stage IV	8	9	9
Received surgery	4	7	6
Received chemotherapy	11	10	10
Received radiation therapy	10	8	7
Albumin	3	3	3
CRP	1	1	2
NLR	2	2	1
PLR	6	11	11

*Note*: The numerical values represent the importance rankings of the features, with 1 indicating the highest rank (i.e., greatest importance as a predictor).

Abbreviations: BMI, body mass index; CRP, C‐reactive protein; NLR, neutrophil–lymphocyte ratio; PLR, platelet lymphocyte ratio.

## 4 Discussion

### 4.1 Prevalence of Depression Among Pancreatic Cancer Patients

At baseline, 35% of pancreatic cancer patients exhibited clinically significant depression (PHQ‐9 ≥ 10), and this rate decreased to around 20% at follow‐up time‐points. This high prevalence of depression aligns with previous studies reporting higher depression rates in pancreatic cancer compared to other cancers [5, 7]. The pattern is consistent with reports that distress peaks around diagnosis and subsequently declines in cancer populations [23, 24], including pancreatic cancer [11, 25]. To evaluate whether survival bias might contribute to the observed trend, we ran a sensitivity analysis on the 163 patients who survived until Month 12. The decrease in the rate of depression after baseline was still observed in this population; the proportion of patients with PHQ‐9 ≥ 10 dropped from 32.9% at baseline to 19.6% at Month 12. We further compared baseline PHQ‐9 scores between survivors and non‐survivors, finding no significant differences (Mann–Whitney *U* = 3367.50, *p* = 0.598; *t* = 0.965, *p* = 0.336). Taken together, these findings imply that survival bias alone is unlikely to explain the decline in depression prevalence. Instead, the improvement in the depression rate is likely to at least partly reflect treatment effects (e.g., symptom relief, better pain control) or natural psychological adaptation as patients adjust to the illness trajectory.

Given the high prevalence of depression and its negative effects on patient outcomes, emerging studies suggest that timely detection and treatment of depression could be beneficial in the care of pancreatic cancer patients [9, 11, 12, 26, 27]. The most effective method to screen patients for depression is a brief screening questionnaire (several options are available) [9], and it is highly recommended that patients receive routine repeated screenings [26, 27]. Once depression is identified in a patient, appropriate treatment can potentially improve quality of life [9]. Groundbreaking findings from a cohort study have also suggested that integrating a mental health professional into the care team for pancreatic cancer patients can significantly decrease mortality rates [11]. This striking finding suggests that mental health care is a vital component of comprehensive multidisciplinary treatment. However, various factors such as the medical environment and patient perception contribute to the low response rate to depression screening questionnaires among cancer patients. As a result, the use of routine patient test indicators as biomarkers to predict depression becomes highly important. Implementing such a system would provide an opportunity to monitor and further confirm the emotional state of patients.

### 4.2 Associations Between Depression and Demographic or Clinical Variables

The risk factors for depression among cancer patients remain largely unclear, and inconsistent results have been reported from studies on pancreatic cancer [28]. In particular, studies on the relationship between depression and age in pancreatic cancer patients have yielded mixed findings. While some research indicates higher depression rates in older patients, others suggest a trend toward depression in younger individuals [9, 25]. Contrary to earlier studies on pancreatic cancer patients that suggested males are more likely to experience depression, recent findings indicate a correlation between females and depression [29]. Other studies, including this one, did not identify any association between depression and patient sex, potentially due to sample size, sex distribution of participants, or the methods used to evaluate depression [25]. Nevertheless, the results from different ML models in this study suggest that gender and age may be important predictors (Table 6).

Our initial univariate GEE studies showed that not undergoing surgical resection and having advanced stage disease were significantly linked to depression in pancreatic cancer patients. These correlations are well aligned with previous findings that advanced disease and the absence of surgical intervention are associated with higher depression rates and increased initiation of antidepressant use [9, 25]. There are several reasons that surgical intervention may not be performed on pancreatic cancer patients. Often, pancreatic cancer is diagnosed at an advanced stage, making surgery impractical due to extensive spread of the tumor. The tumor location may also complicate surgical removal if it involves critical structures like major blood vessels. Additionally, the patient's overall health and existing comorbidities might render them unfit for surgery. Sometimes, tumors do not sufficiently respond to preoperative treatments such as chemotherapy or radiation, which can preclude surgical interventions. Finally, personal preferences may lead some patients to opt out of surgery, as the patient may favor less invasive treatments or palliative care instead. All of these factors influence the clinical decision‐making process regarding treatment of pancreatic cancer with surgical interventions. Consequently, these factors may contribute to a psychological burden and result in higher inflammatory markers for patients, reinforcing the connection between poor prognosis and depression as demonstrated in earlier studies [6, 30].

### 4.3 Associations Between Depression and Inflammatory Markers

In this study, we found that inflammatory markers were significantly correlated with depression in pancreatic cancer patients. Moreover, ML identified CRP as the most important predictor of depression. This observation mirrors trends seen in studies of lung cancer patients, where the association between depression and CRP was more pronounced than that of depression and albumin, suggesting that CRP might be a more definitive marker of depression in such cohorts [31]. Tandem alterations in albumin and CRP levels can bolster clinical diagnosis of inflammation and support the predictive power of acute‐phase reactants in the identification of depressive symptoms among cancer patients.

Calculated values of NLR and PLR from routine blood counts can serve as cost‐effective markers for systemic inflammation and may hold prognostic value for various cancers, including pancreatic cancer [32]. The association of these markers with depression has been extensively studied, but the studies have yielded mixed results [33]. In our analyses, both NLR and PLR showed significant associations with depression in pancreatic cancer patients. However, only the association with NLR persisted after more in‐depth multivariate analysis, aligning with recent evidence that NLR is an independent predictor of depression in this population [34]. Such findings suggest that while both NLR and PLR could be elevated in depressive states, NLR may be a more reliable indicator of depression [33, 35]. The contrasting results from different analyses and in different studies highlight the complexity of the relationships between these markers and depression. Thus, further research will be needed, and a particular focus on PLR is warranted.

To test whether inflammation remains associated with depressive symptoms in patients with advanced disease, we conducted a subgroup analysis limited to stage IV patients (*n* = 180). The data for each inflammatory marker, including log‐transformed CRP, NLR, PLR, and albumin, were stratified according to the median baseline value. Patients with high CRP, NLR, or PLR had significantly higher baseline PHQ‐9 scores than those with lower levels (*p* < 0.05 for each marker), while albumin levels showed no significant association with depression. These findings underscore the link between inflammation and depression, even in individuals with advanced disease.

### 4.4 Predictive Machine Learning Models for Depression

This study demonstrates the feasibility of applying ML models to utilize routinely collected clinical and inflammation biomarker data for prediction of clinically significant depression (PHQ‐9 ≥ 10) in patients with pancreatic cancer. The performance characteristics of all four evaluated models were comparable. AUC values ranged from 0.70 to 0.72, indicating a moderate but consistent level of discriminative ability.

Although no single model outperformed the others by a wide margin, XGBoost and SVM had the highest macro F1‐scores (0.63), suggesting these two had slightly better balance between sensitivity and specificity under class imbalance. RF had the highest specificity (0.75), indicating its potential to minimize false positives. These results underscore the value of ensemble tree‐based and margin‐based methods in modeling complex psychiatric outcomes from heterogeneous clinical features.

Previously Abdulla et al. [36] demonstrated that eight‐analyte cytokine panels could be analyzed with the XGBoost model to classify depression severity in a diabetes‐screening cohort (AUC 0.95; accuracy 0.89). Consistent with their findings, our results confirm that peripheral inflammatory signatures are informative of depression status, even in a far more medically complex setting. By analyzing the results of inexpensive routine tests (CRP, NLR, PLR, and albumin) from 328 pancreatic cancer patients with longitudinal tracking for up to 12 months, we demonstrate that reliable but clinically realistic performance (AUC 0.70–0.72; macro‐F1 0.63) can be achieved for prediction of depression. Moreover, our repeated‐measures design captures within‐patient dynamics, and the inclusion of full confusion matrices and feature‐importance outputs enhances interpretability. Together, these advances can help to extend biomarker‐based mental health screening from metabolic clinics to oncology wards, laying the groundwork for cost‐effective, real‐time monitoring of depression in routine cancer care.

This study is among the first to apply ML models to analyze inflammatory markers for the detection of depression in pancreatic cancer. Although the performance of our models is not yet sufficient for clinical utilization, this study offers a valuable foundation for future research. With larger and more diverse datasets, predictive accuracy and generalizability could be further enhanced, supporting the potential role of ML‐based tools in early psychosocial risk stratification in oncologic care. Our models exclusively utilize structured clinical and laboratory data already present in EHR databases. Thus, the analyses are inherently compatible with routine care and can plausibly be deployed with minimal additional data entry. Similar to the successful integration of depression‐risk screening demonstrated by Nickson et al. [37], our algorithms could be implemented in the background, providing real‐time analysis of inflammatory markers with demographic and clinical variables to identify patients with elevated risk of depression and trigger automated alerts for early psychological assessment or referral. While the present work provides a proof of concept for this type of application, it will be necessary to perform rigorous prospective validation across multiple centers, along with impact analyses to weigh benefits against alert fatigue and resource use. Close collaborations with hospital IT departments and clinicians will also be necessary for a potential clinical rollout.

Limitations of this study include the modest sample size and predominance of stage IV cases, which limits confidence in applying our findings to patients with earlier stage pancreatic cancer. Future studies should recruit sufficient numbers of early‐stage patients and test the models in larger, stage‐balanced cohorts to confirm their broader utility. Moreover, our study cohort consisted exclusively of patients with pancreatic cancer, a population characterized by advanced disease, profound systemic inflammation, and distinctive psychosocial stressors, which inevitably constrains the generalizability of our findings to other conditions. Cancer‐specific biology and treatment regimens can modulate both inflammatory cascades and mood‐related pathways. For instance, breast‐cancer survivors often experience long survivorship periods with endocrine therapy‐related symptoms, whereas lung‐cancer patients face rapid functional decline and exhibit different cytokine profiles. Consequently, the strength and direction of associations between inflammatory biomarkers and depressive symptoms are expected to differ for different malignancies. To determine whether the strategy of biomarker‐based risk stratification proposed here has broader clinical utility, it will be essential to perform studies in diverse cancer populations, such as breast, lung, and colorectal cohorts with varying stages and treatment exposures. Multi‐center prospective studies that integrate tumor type‐specific variables, longitudinal inflammatory trajectories, and nuanced psychological assessments may shed light on whether shared or unique mechanistic pathways underpin depression across cancers. Such efforts can also inform whether a universal screening algorithm is feasible or whether tailored, tumor‐specific models are required to optimize early detection and intervention for cancer‐related depression. Another limitation of this study is that there were potential inconsistencies in the timing and frequency of data collection. The study relied on specific inflammatory markers (CRP, NLR, and PLR) and self‐reported measures of depression, which may introduce biases. Depressive symptoms often go unnoticed in oncology settings because clinicians have few practical tools for systematic screening, potentially due to limited psychiatric training, intense time pressures, and the complexity of cancer care [38]. In light of these constraints, the nine‐item PHQ‐9 is a feasible and cost‐effective screening tool, and international guidelines from ESMO, NICE, and ASCO recommend its routine use in cancer settings. Integrating the PHQ‐9 into oncology workflows can enable rapid identification and triage of patients who may require formal psychiatric assessments, thereby promoting earlier intervention and potentially improving outcomes [27, 39]. In our large pancreatic cancer cohort (assembled entirely from routinely captured EHR data), the PHQ‐9 provided an optimal balance of practicality, standardization, and real‐world relevance. Nonetheless, reliance on a self‐report screening test rather than a structured diagnostic interview remains an important study limitation. We used a standard PHQ‐9 cut‐off (≥ 10) to define clinically significant depression, aligning with common oncology screening practices. While this binary approach supports clinical actionability, future studies should model PHQ‐9 as a continuous or ordinal variable to capture the degree of symptoms. A key limitation of our models is the exclusion of dedicated psychological covariates, such as pre‐existing psychiatric diagnoses, socioeconomic status, and stress‐related exposures; this information was not consistently available in the source data. Prospective studies should therefore collect standardized mental‐health histories, socioeconomic metrics, and validated stress measures to disentangle the potential influence of these factors. Incorporating such information would strengthen confounder control, enhance interpretability, and allow for more comprehensive biopsychosocial risk stratification. Finally, the study was limited by concurrent collection of inflammatory markers and depression data. Therefore, the models reflect diagnostic classifications rather than prospective predictions. Future studies should adopt temporally staggered designs to enable forecasting of depression.

Our study highlights the critical interplay between inflammation and depression in pancreatic cancer patients, emphasizing the need for integration of mental health evaluation into cancer care. Utilizing inflammatory markers for depression risk assessment enables early detection and personalized treatment strategies, while ML algorithms enhance diagnostic precision and treatment optimization. These findings underscore the importance of continued research to improve prediction and management strategies, supporting a proactive, patient‐centered approach in oncology.

## Conflicts of Interest

The authors declare no conflicts of interest.

## Data Availability

The data that support the findings of this study are available on request from the corresponding author. The data are not publicly available due to privacy or ethical restrictions.

## References

[1] B. Jagadeesan , P. H. Haran , D. Praveen , P. R. Chowdary , and M. V. Aanandhi , “A Comprehensive Review on Pancreatic Cancer,” Research Journal of Pharmacy and Technology 14 (2021): 552–554.

[2] F. Hand and K. C. Conlon , “Pancreatic Cancer,” Surgery (Oxford) 37 (2019): 319–326.

[3] H. Sung , J. Ferlay , R. L. Siegel , et al., “Global Cancer Statistics 2020: GLOBOCAN Estimates of Incidence and Mortality Worldwide for 36 Cancers in 185 Countries,” CA: A Cancer Journal for Clinicians 71 (2021): 209–249. 33538338 10.3322/caac.21660

[4] J. Huang , V. Lok , C. H. Ngai , et al., “Worldwide Burden of, Risk Factors for, and Trends in Pancreatic Cancer,” Gastroenterology 160 (2021): 744–754. 33058868 10.1053/j.gastro.2020.10.007

[5] M. J. Massie , “Prevalence of Depression in Patients With Cancer,” Journal of the National Cancer Institute. Monographs 2004 (2004): 57–71. 10.1093/jncimonographs/lgh014 15263042

[6] T. J. Hartung , E. Brahler , H. Faller , et al., “The Risk of Being Depressed Is Significantly Higher in Cancer Patients Than in the General Population: Prevalence and Severity of Depressive Symptoms Across Major Cancer Types,” European Journal of Cancer 72 (2017): 46–53. 28024266 10.1016/j.ejca.2016.11.017

[7] A. F. Barnes , T. P. Yeo , B. Leiby , A. Kay , and J. M. Winter , “Pancreatic Cancer‐Associated Depression: A Case Report and Review of the Literature,” Pancreas 47 (2018): 1065–1077. 30199487 10.1097/MPA.0000000000001148

[8] L. Jia , S. M. Jiang , Y. Y. Shang , et al., “Investigation of the Incidence of Pancreatic Cancer‐Related Depression and Its Relationship With the Quality of Life of Patients,” Digestion 82 (2010): 4–9. 20145402 10.1159/000253864

[9] C. A. Boyd , J. Benarroch‐Gampel , K. M. Sheffield , Y. Han , Y. F. Kuo , and T. S. Riall , “The Effect of Depression on Stage at Diagnosis, Treatment, and Survival in Pancreatic Adenocarcinoma,” Surgery 152 (2012): 403–413. 22938900 10.1016/j.surg.2012.06.010 PMC3465163

[10] N. Sato , Y. Hasegawa , A. Saito , et al., “Association Between Chronological Depressive Changes and Physical Symptoms in Postoperative Pancreatic Cancer Patients,” Biopsychosocial Medicine 12 (2018): 13. 30288172 10.1186/s13030-018-0132-1 PMC6162953

[11] T. Seoud , A. Syed , N. Carleton , et al., “Depression Before and After a Diagnosis of Pancreatic Cancer: Results From a National, Population‐Based Study,” Pancreas 49 (2020): 1117–1122. 32833946 10.1097/MPA.0000000000001635 PMC7450395

[12] N. E. Davis , J. J. Hue , R. K. Kyasaram , et al., “Prodromal Depression and Anxiety Are Associated With Worse Treatment Compliance and Survival Among Patients With Pancreatic Cancer,” Psychooncology 31 (2022): 1390–1398. 35470512 10.1002/pon.5945

[13] P. S. Chen , Y. C. Shen , C. F. Lin , et al., “Prognostic Impacts of Depression and Inflammatory Factors in Pancreatic Cancer Patients,” Biopsychosocial Science and Medicine 87 (2025): 146–152. 39909013 10.1097/PSY.0000000000001368

[14] B. J. Kenner , “Early Detection of Pancreatic Cancer: The Role of Depression and Anxiety as a Precursor for Disease,” Pancreas 47 (2018): 363–367. 29498966 10.1097/MPA.0000000000001024 PMC5865482

[15] W. Breitbart , B. Rosenfeld , K. Tobias , et al., “Depression, Cytokines, and Pancreatic Cancer,” Psychooncology 23 (2014): 339–345. 24136882 10.1002/pon.3422 PMC4220448

[16] N. Mac Giollabhui , T. H. Ng , L. M. Ellman , and L. B. Alloy , “The Longitudinal Associations of Inflammatory Biomarkers and Depression Revisited: Systematic Review, Meta‐Analysis, and Meta‐Regression,” Molecular Psychiatry 26 (2021): 3302–3314. 32807846 10.1038/s41380-020-00867-4 PMC7887136

[17] S. W. Cole , A. S. Nagaraja , S. K. Lutgendorf , P. A. Green , and A. K. Sood , “Sympathetic Nervous System Regulation of the Tumour Microenvironment,” Nature Reviews. Cancer 15 (2015): 563–572. 26299593 10.1038/nrc3978 PMC4828959

[18] A. Eckerling , I. Ricon‐Becker , L. Sorski , E. Sandbank , and S. Ben‐Eliyahu , “Stress and Cancer: Mechanisms, Significance and Future Directions,” Nature Reviews. Cancer 21 (2021): 767–785. 34508247 10.1038/s41568-021-00395-5

[19] Y. Sánchez‐Carro , A. de la Torre‐Luque , I. Leal‐Leturia , et al., “Importance of Immunometabolic Markers for the Classification of Patients With Major Depressive Disorder Using Machine Learning,” Progress in Neuro‐Psychopharmacology & Biological Psychiatry 121 (2023): 110674. 36332700 10.1016/j.pnpbp.2022.110674

[20] P. Cruz‐Gonzalez , A. W.‐J. He , E. P. Lam , et al., “Artificial Intelligence in Mental Health Care: A Systematic Review of Diagnosis, Monitoring, and Intervention Applications,” Psychological Medicine 55 (2025): e18. 39911020 10.1017/S0033291724003295 PMC12017374

[21] A. de Hond , M. van Buchem , C. Fanconi , et al., “Predicting Depression Risk in Patients With Cancer Using Multimodal Data: Algorithm Development Study,” JMIR Medical Informatics 12 (2024): e51925. 38236635 10.2196/51925 PMC10835583

[22] P. Thekkumpurath , J. Walker , I. Butcher , et al., “Screening for Major Depression in Cancer Outpatients: The Diagnostic Accuracy of the 9‐Item Patient Health Questionnaire,” Cancer 117 (2011): 218–227. 20737537 10.1002/cncr.25514

[23] W. Linden , A. Vodermaier , R. Mackenzie , and D. Greig , “Anxiety and Depression After Cancer Diagnosis: Prevalence Rates by Cancer Type, Gender, and Age,” Journal of Affective Disorders 141 (2012): 343–351. 22727334 10.1016/j.jad.2012.03.025

[24] A. M. Krebber , L. M. Buffart , G. Kleijn , et al., “Prevalence of Depression in Cancer Patients: A Meta‐Analysis of Diagnostic Interviews and Self‐Report Instruments,” Psychooncology 23 (2014): 121–130. 24105788 10.1002/pon.3409 PMC4282549

[25] K. E. Dengsø , E. W. Andersen , T. Thomsen , et al., “Increased Psychological Symptom Burden in Patients With Pancreatic Cancer: A Population‐Based Cohort Study,” Pancreatology 20 (2020): 511–521. 31973981 10.1016/j.pan.2020.01.001

[26] N. Akizuki , K. Shimizu , M. Asai , et al., “Prevalence and Predictive Factors of Depression and Anxiety in Patients With Pancreatic Cancer: A Longitudinal Study,” Japanese Journal of Clinical Oncology 46 (2016): 71–77. 26590013 10.1093/jjco/hyv169

[27] L. Del Piccolo , V. Marinelli , M. A. Mazzi , et al., “Prevalence of Depression in a Cohort of 400 Patients With Pancreatic Neoplasm Attending Day Hospital for Major Surgery: Role on Depression of Psychosocial Functioning and Clinical Factors,” Psychooncology 30 (2021): 455–462. 33247996 10.1002/pon.5607

[28] A. Pitman , S. Suleman , N. Hyde , and A. Hodgkiss , “Depression and Anxiety in Patients With Cancer,” BMJ (Clinical Research Ed) 361 (2018): k1415. 10.1136/bmj.k1415 29695476

[29] J. C. Holland , A. H. Korzun , S. Tross , et al., “Comparative Psychological Disturbance in Patients With Pancreatic and Gastric Cancer,” American Journal of Psychiatry 143 (1986): 982–986. 3524279 10.1176/ajp.143.8.982

[30] S. D. Passik and W. S. Breitbart , “Depression in Patients With Pancreatic Carcinoma,” Diagnostic and Treatment Issues. Cancer 78 (1996): 615–626. 8681300 10.1002/(SICI)1097-0142(19960801)78:3<615::AID-CNCR42>3.0.CO;2-Z

[31] D. C. McFarland , W. Breitbart , A. H. Miller , and C. Nelson , “Depression and Inflammation in Patients With Lung Cancer: A Comparative Analysis of Acute Phase Reactant Inflammatory Markers,” Psychosomatics 61 (2020): 527–537. 32331769 10.1016/j.psym.2020.03.005 PMC7529727

[32] J. J. Yang , Z. G. Hu , W. X. Shi , T. Deng , S. Q. He , and S. G. Yuan , “Prognostic Significance of Neutrophil to Lymphocyte Ratio in Pancreatic Cancer: A Meta‐Analysis,” World Journal of Gastroenterology 21 (2015): 2807–2815. 25759553 10.3748/wjg.v21.i9.2807 PMC4351235

[33] M. Su , X. Ouyang , and Y. Song , “Neutrophil to Lymphocyte Ratio, Platelet to Lymphocyte Ratio, and Monocyte to Lymphocyte Ratio in Depression: A Meta‐Analysis,” Journal of Affective Disorders 308 (2022): 375–383. 35439466 10.1016/j.jad.2022.04.038

[34] J. Song , D. H. Hong , S. Park , et al., “Association of Inflammation With Depression in Pancreatic Ductal Adenocarcinoma,” Journal of Clinical Oncology 43 (2025): e24059‐e.

[35] H. Kinoshita , D. Takekawa , T. Kudo , K. Sawada , T. Mikami , and K. Hirota , “Higher Neutrophil‐Lymphocyte Ratio Is Associated With Depressive Symptoms in Japanese General Male Population,” Scientific Reports 12 (2022): 9268. 35661149 10.1038/s41598-022-13562-x PMC9166769

[36] H. Abdulla , M. Maalouf , and H. F. Jelinek , “Machine Learning for the Prediction of Depression Progression From Inflammation Markers,” Annual International Conference of the IEEE Engineering in Medicine and Biology Society 2023 (2023): 1–4. 10.1109/EMBC40787.2023.10340436 38082683

[37] D. Nickson , C. Meyer , L. Walasek , and C. Toro , “Prediction and Diagnosis of Depression Using Machine Learning With Electronic Health Records Data: A Systematic Review,” BMC Medical Informatics and Decision Making 23 (2023): 271. 38012655 10.1186/s12911-023-02341-x PMC10680172

[38] W. Sollner , A. DeVries , E. Steixner , et al., “How Successful Are Oncologists in Identifying Patient Distress, Perceived Social Support, and Need for Psychosocial Counselling?,” British Journal of Cancer 84 (2001): 179–185. 11161373 10.1054/bjoc.2000.1545 PMC2363697

[39] L. Grassi , R. Caruso , M. B. Riba , et al., “Anxiety and Depression in Adult Cancer Patients: ESMO Clinical Practice Guideline,” ESMO Open 8 (2023): 101155. 37087199 10.1016/j.esmoop.2023.101155 PMC10163167

